# Supplementary material for: Status of Infectious Diseases in Free-Ranging European Brown Hares (Lepus europaeus) Found Dead between 2017 and 2020 in Schleswig-Holstein, Germany
Source: Pathogens. 2023 Feb 2;12(2):239. doi: 10.3390/pathogens12020239 (PMC9959346; doi:10.3390/pathogens12020239)
Supplement: Supplementary file 1 [file pathogens-12-00239-s001.zip › pathogens-2163462-supplementary.pdf]

Supplements

Lagomorpha-dissection protocol

Dissection team: ……………………..

…………………………………………

Diss.-Nr:…………………………… **Spec.**:……………………………….. Total length:…………………..cm
Date of finding:…..…………………. Dissection date;……………...……. Weight:………………………..g
Location of finding:…………………. Sex: ☐ male ☐ female
Finder:………………………………

Origin: ☐ Hunted ☐ Deceased ☐ Road kill

Age: (Sign of Stroh) ☐ positive (juvenil) ☐ negative (adult) ☐ uncertain

Decomp. Grade ☐ (1-5) ☐ unfrozen ☐ frozen

☐ Photo

Anamnesis:

Sampling day:
☐ Serum [ml]
☐ Plasma [ml]
☐ Eyes (ri le)
☐ Urine sample
☐ Additional

**Measurements [cm]**		
Axillary girth [AU]:		
Total lenght (TL): Dorsal recumbency, neck stretched, nose tip till anus		
Ear length (OL)		
Hindfood length (HL) plantar, without claws		
Tail length (SL) Tail tip until anus		
**Organ weights [g]**		
Liver:		
Spleen:		
Heart (frinse first):		
Brain:		
Retro. Fat:		
Kidney:	le:	ri:
Adrenal gland:	le:	ri:
Thyroid:	le:	ri:
Testis + epididymal:	le:	ri:
Testis w/o epididymal:	le:	ri:
Ovaries:	le:	ri:

**Measurements Uterus/Ovaries/Testes [cm]**			
Ovary (ri):	L:	W:	H:
Ovary (le):	L:	W:	H:
Uterus (diameter; change to bifurcation):			
Placental scars:	le:	ri:	
Testis + epididymal (ri):	L:	W:	H:
Testis + epididymal (le):	L:	W:	H:
Testis w/o epididymal (ri):	L:	W:	H:
Testis w/o epididymal (le):	L:	W:	H:

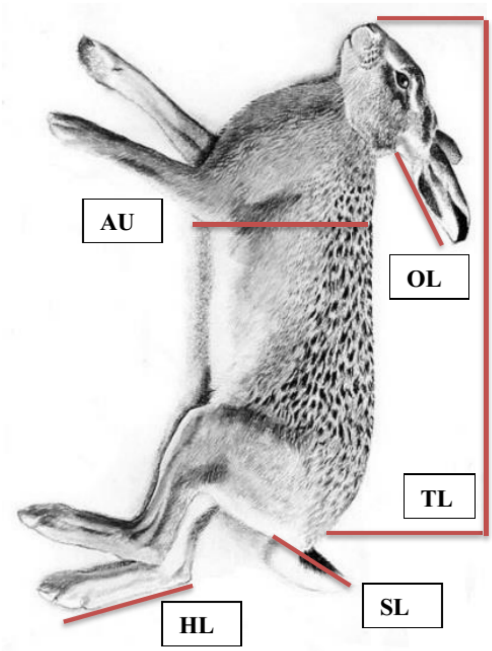

Diss.-Nr:

Nutritional status: ☐ good ☐ moderate ☐ poor
Musculature: ☐ good ☐ moderate ☐ poor
Retro. fat: ☐ good ☐ moderate ☐ poor

Dental abrasion:

Urinary bladder: ☐ full ☐ empty ☐ urine collected

**Parasites:**					
Lung (rinse over sieve)	None ☐				
	Location:	Bronchi	mild ☐	mod. ☐	sev. ☐
		Vessels	mild ☐	mod. ☐	sev. ☐
Heart (rinse over sieve)	None ☐				
	Location:	ri. Atrium	mild ☐	mod. ☐	sev. ☐
		ri. Ventricle	mild ☐	mod. ☐	sev. ☐
		le. Atrium	mild ☐	mod. ☐	sev. ☐
		le. Ventricle	mild ☐	mod. ☐	sev. ☐
Stomach (rinse over sieve)	None ☐				
	Location:		mild ☐	mod. ☐	sev. ☐
Intestine	None ☐				
	Location:	Duodenum	mild ☐	mod. ☐	sev. ☐
		Jejunum	mild ☐	mod. ☐	sev. ☐
		Ileum	mild ☐	mod. ☐	sev. ☐
		Caecum	mild ☐	mod. ☐	sev. ☐
		Colon	mild ☐	mod. ☐	sev. ☐
Liver (rinse over sieve)	None ☐				
	Location:		mild ☐	mod. ☐	sev. ☐
Skin/Fur	None ☐				
	Location:		mild ☐	mod. ☐	sev. ☐

Notes:

Diss.-Nr:

Sampling Lagomorpha

**Age determination (formalin 10%; room tempurature)**		**Reproduction (container with H2O; -20°)**	
	Eyes ri + le		Vagina, Uterus
**Parasites (70% Alcohol)**		**Microbiology (swab or tissue, cooled)**	
	Lung		Swab lung
	Stomach		Swab small intestine
	Intestine		Spleen (tissue)
	Liver		Liver (tissue)
	Heart		Kidney (tissue)
	Skin/ Fur		Brain (tissue)
	Faeces (ca. 8g, container, cooled)		Mesent. Lymphn. (tissue)
			Lung
			Small Intestine
**Histo (Formalin 10%)**			
	Lung (6x: both lobes 1x cran, med., caud., respect.)	**Retained samples (plastic bag; -80°C)**	
	Heart		Lung
	Liver (le/ri)		Heart
	Spleen		Liver
	Muscle		Spleen
	Retroperit. Fat		Muscle
	Kidney (le/ri)		Retro. Fat
	Mesent. Lymphn.		Brain
	Small Intestine (Jejunum, Duodenum)		Kidney
	Large Intestine (Caecum, Colon)		Mesent. Lymphn.
	Adrenal glands (le/ri)		Small Intestine
	Thyroid gland		Large Intestine
	Bone marrow (broken femur)		Cranial bone (half)
	Cranial bone (half)		Gonads
	Gonads (testis/ovaries)		
	Thymus	**Dietary analysis (plastic bag, -20°C)**	
	Pancreas		Stomach contents
	Stomach		
	Trachea	**Toxicology (urine container, -80°C)**	
	Aorta		Urine
	Spinal cord		
	Brain		
	A: Liver (ri/le), Kidney (ri/le)		
	B: Spleen, Mesent. Lymphn., Heart, Retro. Fat		
	C: Adrenal (ri/le), Mescle, Small Int., Large Int.		
	D: Brain		
	E: Lung (6)		
	F: Trachea, Aorta, Stomach, Pancreas		
**OIE Italy (EBHS/RHD2)**			
	Serum (-20°) 1 ml		
	Liver (Eppendorf) cooled		
**FLI JENA (Tularemia)**			
	Liver (Eppendorf) cooled		
	Spleen (Eppendorf) cooled		
	Sampling on day of hunt or finding/ when not frozen		

Figure S1: Lagomorpha dissection and sampling protocol.

Table S1: Incidence of pathomorphological findings of European brown hares and the yearly distribution. Total percentages are given only for routinely collected histopathological samples or macroscopic detectable alterations.

Morphological findings	2017	2018	2019	2020	Total	Total %
**Alimentary system**						
Dental displacement				1	1	0.8
Stomatitis	1				1	
Gastric amyloidosis	1			3	4	9.8
Gastritis				1	1	2.4
Gastric haemorrhages	1	1	1		3	7.3
Intestinal amyloidosis			1	4	5	4.5
Intestinal haemorrhages	1			1	2	1.8
Enteritis	23	3	5	17	48	40.7
Intestinal fibrosis			1		1	0.9
Parasites in intestine	36	2	10	19	67	60.4
Hepatic amyloidosis	3			4	7	6.0
Hepatic haemorrhages	1				1	0.9
Hepatic fibrosis	4	1	1	1	7	6.0
Kupffer cell haemosiderosis				1	1	0.9
Hepatitis	13	3	7	15	38	32.8
Hepatolipidosis	1			4	5	4.3
Liver necrosis	2	1	9	3	15	12.9
Hepatic tumour metastasis	1	1			2	1.7
Bile duct proliferation			1	1	2	1.7
Cholangitis				1	1	0.9
Pancreatic amyloidosis	1			1	2	11.1
Pancreatic fibrosis			1		1	5.6
Pancreas neoplasia	1				1	5.6
**Cardiovascular system**						
Vasculitis				3	3	
Thrombosis	1			2	3	
Epicarditis	2			2	4	3.5
Endocardial haemorrhages				1	1	0.9
Endocardial oedema				1	1	0.9
Myocarditis	1			1	2	1.8
Cardial parasitosis	1				1	0.9
Endocardiosis				1	1	0.9
**Abdominal and thoracic cavity**						
Haemoabdomen	2	1		3	6	5.1
Haemothorax		1		3	4	3.4
Steatitis	2	3	2	2	9	20.9
Pericardial steatitis				1	1	0.9
Pleura fibrosis	1	1			2	1.7
Peritonitis	1			1	2	1.7
Pleuritis	2			1	3	2.5

Morphological findings	2017	2018	2019	2020	Total	Total %
Trauma/perforation				6	6	5.1
**Haematopoetic and endocrine system**						
Osteomyelitis				1	1	
Lymph node/ Peyer`s patches hyperplasia	2		1	6	9	8.1
Lymphadenitis	2	2		4	8	7.5
Lymph node necrosis			1		1	0.9
Lymphonodal tumour metastasis		1	1		2	1.9
Lymphonodal sinus histiocytosis				1	1	0.9
Splenic amyloidosis	2			6	8	7.4
Splenic hyperplasia	3			4	7	6.5
Splenic haemosiderosis	10	1	2	3	16	14.8
Splenitis	1	2	1	2	7	5.6
Splenic necrosis				1	1	0.9
Adrenalitis	3	2		1	6	5.5
Adrenal amyloidosis	5			5	10	9.1
Adrenal adenoma		1			1	0.9
Adrenal atrophy	1			1	2	1.8
Adrenal haemorrhages				1	1	0.9
**Musculoskeletal system**						
Mesenchymal tumour	1				1	0.9
Bone fracture	4	2		4	10	8.5
Disarticulation				1	1	0.9
Limb stump			1		1	0.9
Muscular haemorrhages		1		2	3	2.5
Myositis	1	1	1	3	6	5.2
Muscular parasitosis			1		1	0.9
**Respiratory system**						
Pulmonary atelectasis				5	5	4.3
Pulmonary haemorrhages	5	1	3	3	10	8.5
Bronchiectasis	2			3	5	4.3
Bronchitis				3	3	2.6
Pneumonia	10	2	3	11	26	22.2
Lung fibrosis	1				1	0.9
Tumour metastasis	1				1	0.9
Tracheal haemorrhages				5	5	4.2
Tracheitis				7	7	21.9
Diaphragmatic rupture	1			2	3	2.5
**Skin and subcutis**						
Dermatitis	1		1	6	8	
Skin lesion	8			1	9	7.6
Epidermal hyperkeratosis				3	3	
Skin tumour	1				1	0.8
Mastitis				2	2	
Fat tissue necrosis				1	1	
Panniculitis	1				1	

Morphological findings	2017	2018	2019	2020	Total	Total %
Subcutaneous haematoma	10	2	3	5	20	16.9
**Urinary and genital system**						
Pyometra	3				3	
Uterine metastasis	1				1	
Orchitis	1				1	
Renal amyloidosis	4		1	4	9	7.8
Renal fibrosis		1	3	1	5	4.3
Nephritis	8	2	5	7	22	19.1
Nephrohydrosis			2	1	3	2.6
Nephrolithiasis		1	1		2	1.7
Intratubular protein casts	1	1	4	4	10	8.7
Renal metastasis		1			1	0.9
Renal sclerosis			2	1	3	2.6
Tubulonephrosis			1		1	0.9
Tubulonecrosis			3	1	4	3.5
Renal tumour	1				1	0.9
Ovarian tumour	1				1	
Balantitis				1	1	

Table S2: Detected bacterial and fungal microorganisms with regard to their organ localization in deceased hares.

Bacteria/ Fungi	Brain	Heart	Intestine	Kidney	Liver	Lung	Mesentericlymph node	Reproductive system	Sceletal muscle	Skin	Spleen	Trachea	Total
Acinetobacter spp.	1		2	3	5	7	1		1		4		**24**
Aeromonas bestiarum						1							**1**
Aeromonas hydrophila						1		1					**2**
Aeromonas spp.		1	4	5	3	5	1		1		1	1	**22**
Arthrobacter spp.			1										**1**
Bacillus spp.	3		4	3	3	8	5	1	3		2		**32**
Bacteroides ovatus			1										**1**
Buttiauxella gaviniae	1	1	1	2	2	5	1		1		1		**15**
Buttiauxella spp.	1		2		1	1	1						**6**
Candida albicans			4	2	2		1			1			**10**
Candida spp.			1										**1**
Carnobacterium maltaromaticum				1	2	7		1	2	1	1		**15**
Cedecea spp.						1							**1**

**Bacteria/ Fungi**	**Brain**	**Heart**	**Intestine**	**Kidney**	**Liver**	**Lung**	**Mesentericlymph node**	**Reproductive system**	**Sceletal muscle**	**Skin**	**Spleen**	**Trachea**	**Total**
Citrobacter braakii	1		1	1	1	2	1			1	1		**9**
Citrobacter freundii					1								**1**
Citrobacter gillenii						1					1		**2**
Clostridum septicum			2		1	1	1						**5**
Clostridium baratii			1			1							**2**
Clostridium perfringens (non-typed)	3		12	1	3	4	2				2		**27**
Clostridium sordellii			1										**1**
Coliform bacteria						3							**3**
Coryneform bacteria (not determinable)				1				1					**2**
Cronobacter spp.	1				1						1		**3**
Dermacoccus spp.			1										**1**
Elisabethkingia miricola								1					**1**
Enterobacter asburiae						1							**1**
Enterobacter bugandensis	1		1	1	1	1	1				1		**7**
Enterobacter cloacae				1	1	2					2		**6**
Enterobacter spp.			4	1	2	9	1			1	2	1	**21**
Enterococcus faecalis						3							**3**
Enterococcus faecium						1							**1**
Enterococcus spp.				1	1	12		1			1		**16**
Erwinia spp.			1	1	1	3							**6**
Escherichia coli	7	1	72	14	23	67	12	3	4		18		**221**
Escherichia coli var. *haemolytica*			8		1	1	1		3				**14**
Escherichia vulneris									1				**1**
Ewingella americana			2		2	4	1						**9**
Flavobacterium spp.			1										**1**
Fungi (not determinable)			5			1		1					**7**
Fusobacterium spp.						1						1	**2**
Gram-negative rod-shaped bacteria				1	1								**2**
Gram-positive rod-shaped bacteria				1							1		**2**
Hafnia alvei	2		5	3	3	4	2	1			3		**23**
Klebsiella pneumoniae						1							**1**
Klebsiella spp.						3					1		**4**
Kluyvera intermedia	2		1	1	1	2	1				1		**9**

**Bacteria/ Fungi**	**Brain**	**Heart**	**Intestine**	**Kidney**	**Liver**	**Lung**	**Mesentericlymph node**	**Reproductive system**	**Sceletal muscle**	**Skin**	**Spleen**	**Trachea**	**Total**
Lactobacillus raffinolactis						1							**1**
Lactococcus garvieae						3							**3**
Leclercia adecarboxylata	1		3	3	3	2	1						**13**
Lelliottia amnigena			2		2	6	1		1		2		**14**
Listeria monocytogenes					1	1							**2**
Mannheimia granulomatis				1	1	2					1		**5**
Mannheimia spp.						1							**1**
Moellerella wisconsensis					1	1							**2**
Morganella morganii			2			4	1				1		**8**
Mucor spp.			2										**2**
Myroides odoratimimus						1							**1**
Myroides spp.						2							**2**
Non-fermenting, gram-negative bacteria (not determinable)					1	5							**6**
Pantoea agglomerans						2				1			**3**
Pantoea spp.	2	1	3	3	3	22	1		4	1	5		**45**
Pasteurella multocida	1		1		1	7	1				2	1	**14**
Penicillium spp.						1							**1**
Proteus spp.	1			3	5	8	1	2	3		2		**25**
Providencia alcalifaciens						1							**1**
Providencia rettgeri				1		1							**2**
Providencia spp.						1							**1**
Pseudomonas aeruginosa						2			1				**3**
Pseudomonas antarctica						2							**2**
Pseudomonas extremorientalis						1							**1**
Pseudomonas fluorescens						1							**1**
Pseudomonas fragi						2							**2**
Pseudomonas koreensis						6							**6**
Pseudomonas libanensis						1							**1**
Pseudomonas lundensis						2							**2**
Pseudomonas nitroreducens								1	2		2		**5**
Pseudomonas rhodesiae						2							**2**
Pseudomonas spp.	2		14	5	9	49	6	2		2	5	1	**95**
Pseudomonas synxantha										1			**1**
Pseudomonas viridiflava						1							**1**
Rahnella aquatilis	3		12	3	5	12	5	1		2	2	1	**46**

**Bacteria/ Fungi**	**Brain**	**Heart**	**Intestine**	**Kidney**	**Liver**	**Lung**	**Mesentericlymph node**	**Reproductive system**	**Sceletal muscle**	**Skin**	**Spleen**	**Trachea**	**Total**
Raoultella ornithinolytica								1			1		**2**
Raoultella spp.						2							**2**
Serratia fonticola			6	3	2	9			2		3		**25**
Serratia liquefaciens	2		5	4	4	11	4	1			4		**35**
Serratia marcescens								1	1				**2**
Serratia plymuthica			1	1		4							**6**
Serratia proteamaculans						1							**1**
Serratia spp.	1		3	3	5	7	1				1	1	**22**
Sphingobacterium spp.						2							**2**
Staphylococcus spp., coagulase-negative					2	5			3				**10**
Staphylococcus aureus	1	1		4	2	8			4	6	2		**28**
Staphylococcus lutetiensis						1							**1**
Staphylococcus sciuri	1		1	1	1		1						**5**
Staphylococcus spp.	1		1	1		1					1		**5**
Staphylococcus xylosus				1		1	1				1		**4**
Stenotrophomonas maltophilia			1				1						**2**
Stenotrophomonas spp.	1					4					1		**6**
Streptococcus spp., α-haem.	1		9	6	7	20	3	1			4		**51**
Streptococcus spp., β-haem.				1		1							**2**
Streptococcus canis	1			1	1	1	1				1		**6**
Streptococcus gallolyticus				1							1		**2**
Streptococcus spp., γ-haem.	4		7	6	8	16	4	2	1		5		**53**
Yarrowia lipolytica	1		7	2		4	2						**16**
Yeast (not further determined)	1		16	4	5	13	6				4	1	**50**
Yersinia enterocolitica						3							**3**
Yersinia pseudotuberculosis			8	4	6	8	2	1		1	3		**33**
**Total**	**48**	**5**	**242**	**106**	**137**	**439**	**76**	**24**	**38**	**18**	**98**	**8**	**1239**
